# Nasopharyngeal carcinoma: nationwide trends in subtype-specific incidence and survival over 3 decades in a non-endemic area

**DOI:** 10.1007/s00432-023-05547-8

**Published:** 2024-01-29

**Authors:** Jort S. van Velsen, Bert van der Vegt, Boudewijn E. C. Plaat, Johannes A. Langendijk, Chantal C. H. J. Epskamp-Kuijpers, Boukje A. C. van Dijk, Sjoukje F. Oosting

**Affiliations:** 1 Department of Medical Oncology, University Medical Center Groningen, University of Groningen, Hanzeplein 1, PO Box 30.001, 9700 RB Groningen, The Netherlands; 2 Department of Pathology and Medical Biology, University Medical Center Groningen, University of Groningen, PO Box 30.001, 9700 RB Groningen, The Netherlands; 3 Department of Otorhinolaryngology-Head and Neck Surgery, University Medical Center, University of Groningen, PO Box 30.001, 9700 RB Groningen, The Netherlands; 4 Department of Radiation Oncology, University Medical Center Groningen, University of Groningen, PO Box 30.001, 9700 RB Groningen, The Netherlands; 5 PALGA Foundation, De Bouw 123, 3991 SZ Houten, the Netherlands; 6 Department of Epidemiology, University Medical Center Groningen, University of Groningen, PO Box 30.001, 9700 RB Groningen, The Netherlands; 7 Department of Research and Development, Netherlands Comprehensive Cancer Organisation (IKNL), PO Box 19079, 3501 DB Utrecht, The Netherlands

**Keywords:** Nasopharyngeal carcinoma, Epstein–Barr virus, Incidence, Survival, Non-endemic

## Abstract

**Purpose:**

To identify trends in incidence and survival of NPC, subdivided by EBV status and histopathological subtype, over a 30-year period in the Netherlands.

**Methods:**

Anonymized data from the Netherlands Cancer Registry and the Dutch Nationwide Pathology Databank (PALGA) for the period 1989–2018 were linked to identify and classify NPC cases.

**Results:**

Incidence of NPC remained stable, with an annual percentage change (APC) of − 0.2. (95% CI − 0.9; 0.5). EBV testing became routine only in the last decade, the incidence of EBV-positive tumors remained stable over this period (APC 1.2, 95% CI − 1.3; 3.8). An increase in EBV-negative tumors (APC: 7.1, 95% CI 2.5; 11.9) and a decrease in untested tumors were found (APC: − 10.7, 95% CI − 15.7; − 5.7). The incidence of non-keratinizing, differentiated tumors increased (APC: 3.8, (95% CI 2.2; 5.5) while the incidence of other histological subtypes remained stable. Overall survival was better in patients diagnosed after 1998 (hazard ratio 0.8, 95% CI 0.6; 0.9). EBV status, histology, stage, and age were independently associated with relative excess risk of dying, but period of diagnosis was not.

**Conclusion:**

Testing for EBV increased over time, and a stable incidence of EBV-positive NPC over the last 10 years. The rising incidence of non-keratinizing, differentiated NPC mirrors data from the US and suggests a shift in non-endemic regions.

**Supplementary Information:**

The online version contains supplementary material available at 10.1007/s00432-023-05547-8.

## Introduction

Nasopharyngeal carcinoma (NPC) originates from the mucosa of the nasopharynx, most often in the fossa of Rosenmüller. NPC is a rare disease in the Western countries, but it is endemic in specific regions in Southeast Asia. The age standardized incidence rate (ASR) varies between 3–15 per 100,000 in endemic countries, whereas the ASR for western countries is less than 1 per 100,000. NPC is more common in males than in females with a ratio of approximately 5:2 (Bray et al. 2018; Ferlay et al. 2018).

NPC is classified according to World Health Organization (WHO) morphological subtypes in: keratinizing carcinoma (WHO type I), and non-keratinizing carcinoma, subdivided in differentiated (WHO type II) and undifferentiated (WHO type III) non-keratinizing carcinomas. A rare type of NPC is the basaloid subtype. Non-keratinizing tumors comprise > 95% of NPC cases in endemic areas, and about half in non-endemic regions. Non-keratinizing tumors are associated with Epstein–Barr virus (EBV), dietary factors and Southeast Asian descent (Pathmanathan et al. 1995; Huang et al. 2021; Young & Dawson 2014), while keratinizing NPCs are associated with tobacco exposure and alcohol consumption (Guo et al. 2009; Tsao et al. 2014; Liu et al. 2016; Chang et al. 2017).

Studies on the genetic susceptibility for NPC have identified certain genetic variations of the HLA genes and TERT/CLPTM1L genes to be associated with a predisposition to NPC (WHO type III) (Bei et al. 2016; Bei et al. 2010; Liu et al. 2017).

Clinical symptoms mainly correlate with tumor invasion of an anatomical region. Common symptoms are a combination of enlarged cervical lymph nodes, often as a first sign of disease, epistaxis, pain, nasal obstruction, hearing loss, and impaired cranial nerve function. NPC usually spreads to regional cervical lymph nodes but can also metastasize to distant sites including lung, liver, and bone. Prognostic factors are stage, histological subtype, age, comorbidities, performance status, and for EBV-related NPC, plasma EBV DNA levels (Lee et al. 2021).

For early-stage disease, radiotherapy alone is the standard of care and for locally advanced disease, chemoradiotherapy with or without neoadjuvant or adjuvant chemotherapy is recommended (Colevas et al. 2018).

Five-year disease-specific survival rates equal approximately 85% for stage I and II disease, 71% for stage III to Iva, and 40% for stage IVb/IVc (Huang et al. 2017).

The incidence and mortality of NPC have decreased in many endemic areas (Tang et al. 2016), but less is known about incidence and survival over time in non-endemic regions, especially from the last decade (Tang et al. 2016; Arnold et al. 2013; Anandan et al. 2008).

Due to the decrease in smoking in the general population, it is conceivable that the incidence of non-EBV-related NPC is falling, whereas EBV-related NPC may have become more prevalent due to immigration and globalization. A study that was published 10 years ago showed an overall decline in NPC incidence in the Netherlands up to 2009 but a significant rise in the subset of non-keratinizing NPC. However, EBV status was not included in that analysis (Arnold et al. 2013).

Over the last decades, the addition of chemotherapy to radiation significantly improved survival (Al-Sarraf et al. 1998). Moreover, radiotherapy techniques have become more sophisticated, and multidisciplinary supportive care has evolved to maximally support the patient through the treatment journey, which could have translated in better overall survival.

The aim of this study was to analyze national trends in incidence and survival of NPC in the Netherlands in general as well as for EBV-related and non-EBV-related disease over a 30-year period.

## Methods

**Fig. 1:**
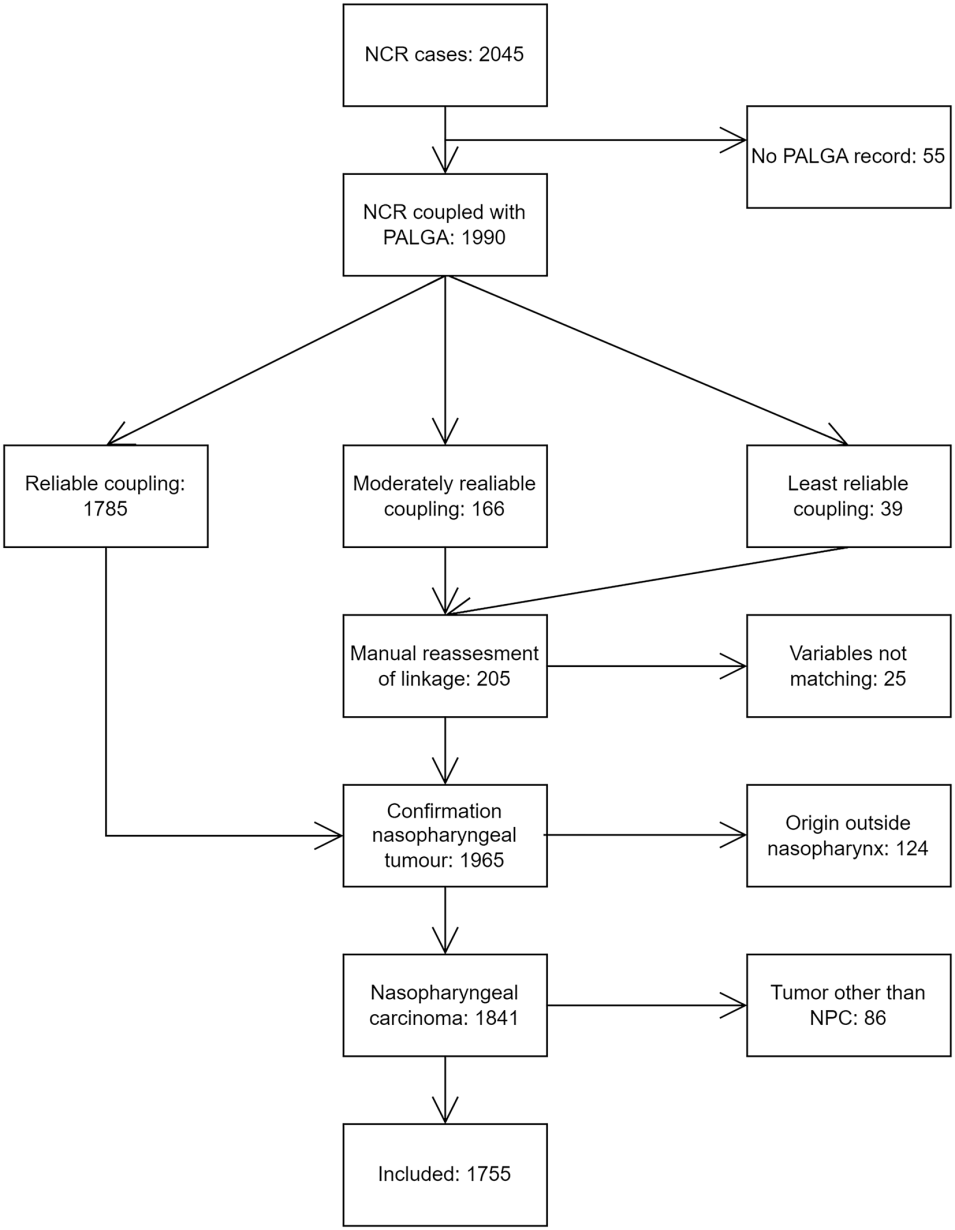
Study diagram. The starting number represents all cases of nasopharyngeal cancer (NPC) in the Netherlands Cancer Registry (NCR) diagnosed between 1989 and 2018

This is a historical observational cohort study. We used the nationwide population-based prospective Netherlands Cancer Registry, which contains all newly diagnosed cancers. The main signaling sources are pathology records obtained from nationwide network and registry of histopathology and cytopathology (PALGA), but yearly also discharge hospital records are used to include only clinically confirmed cancers. Information on patient, tumor, and treatment characteristics are collected from hospital records by trained data managers including the applicable TNM classification and stage at the time of diagnosis (for an overview of the TNM classifications used during the time period, see Table S1, Appendix A). Excerpts of pathology reports of Dutch hospitals are collected in PALGA (Casparie et al. 2007). Data from the Netherlands Cancer Registry on all NPC cases from January 1989 to January 2019 were linked to PALGA by a trusted third party based on year of birth, year of diagnosis, gender, and diagnosis of NPC. In total, 2045 records were retrieved from the Netherlands Cancer Registry over the period 1989–2018. Out of these, 55 cases could not be linked to PALGA data (Fig. 1). The linked cases were divided in three categories based on reliability of linkage. All moderately reliable and least reliable linked records were judged case by case to decide if records were linked correctly or not. Reliability was checked by comparing year of diagnosis, age, sex, and diagnosis. Next, pathological information from PALGA was reviewed for every case to determine consistency with a diagnosis of NPC. 123 cases were deemed to have another origin than the nasopharynx. Furthermore, 86 cases of tumors arising from the nasopharyngeal region, but of non-epithelial origin, were excluded. The final number of cases included in this study was 1755.

With the information from PALGA, data on the origin of the biopsies (primary tumor, lymph node or distant metastasis), the histological subgroup according to WHO classification, EBV status, and differentiation grade were collected or deduced. WHO classifications were assigned based on the following terms in the pathology report: keratinization or keratin positive or verrucous for WHO type I; non-keratinizing, but other histopathological signs of squamous cell carcinoma, e.g., cell bridges for WHO type II, and undifferentiated or poorly differentiated for WHO type III, but also alternative descriptions including lymphoepithelial carcinoma, lymphoepithelioma, Schmincke’s tumor, Regaud’s tumor, and spindle cell carcinoma were included in WHO type III. All cases where alternative descriptions were used were discussed with a trained pathologist. Tumors which could not be classified as WHO type I, II, III or as basaloid were assigned “not otherwise specified” (NOS). Vital status was provided by the Netherlands Cancer Registry, based on the linkage to municipal records on February 1st, 2020. The date of death, date of removal from municipal records or the linkage date was used to calculate the follow-up time. Survival time was defined as the time between diagnosis and date of death and was censored at the date of removal from municipal records or the last date of linkage with municipal records. To assess changes over time, year of diagnosis was recorded and grouped in 5-year periods to ensure sufficient numbers for comparison. Furthermore, survival time by year group was subdivided by EBV status and by pathological classification.

### Statistical analysis

For the primary outcome, incidence over time, we first counted the number of new cases and calculated the European standardized incidence rate (ESR) of NPC, using population data for the Netherlands from the Central Bureau of Statistics (CBS) and the European standard population established in 1976 (Waterhouse et al. 1976). This ensures that incidence rates are comparable, even when there are differences in population size and age distribution over time. Then we used join point regression (Join point Regression Program, Version 4.8.0.1-April 2020; Statistical Methodology and Applications Branch, Surveillance Research Program, National Cancer Institute, Maryland, USA), to assess the trend and the existence of trend breaks in log-transformed ESR over time. We calculated the annual percentage change (APC) per year with the corresponding 95% confidence interval and assessed the existence of trend breaks using permutation tests (Kim et al. 2000). This information showed whether the trend was increasing, decreasing or stable (Kim et al. 2000). These analyses were repeated for EBV-positive, EBV-negative, and EBV status unknown subgroups. We additionally calculated trends in incidence over time for each histological subgroup.

Follow-up time was calculated as the number of years between the date of diagnosis and the date of death, emigration, or censoring date (February 1, 2020). Kaplan–Meier survival analyses were used to estimate median survival for the groups included in the trend analysis. The differences between the survival curves were tested using the log-rank test. Cox regression analyses were performed to allow for a multivariable analysis on year groups and EBV status, after checking whether the proportional hazard assumption was met by evaluating proportionality of categories using log minus log plots (LML-plots). For the analysis, IBM SPSS was used (IBM Corp. Released 2020. IBM SPSS Statistics for Windows, Version 27.0. Armonk, NY: IBM Corp). Relative survival rates were calculated using the Ederer II method for relative survival (Dickman & Coviello 2015). In brief, this is the ratio of the observed survival rate compared with the expected survival rate (based on sex, age and calendar year number from Statistics Netherlands (CBS)). Poisson regression modeling was used to calculate relative excess risk of death (Dickman et al. 2004). Statistical analyses were performed using Stata/SE 17.0. Figures were generated using GraphPad Prism version 9.0.0 for Windows, GraphPad Software, San Diego, California USA and IBM SPSS.

## Results

### Patients

The majority of the patients were male (72%) and had locoregionally advanced disease at diagnosis (Table 1). The most prevalent histological subclass was WHO type III (56%), followed by WHO type II (20%). The patient and tumor characteristics of the 1755 included subjects are shown in Table 1, and for subgroups according to EBV status and year of diagnosis in Table 2. We observed a rise in absolute numbers of test results for EBV over time, and a decrease in the number of tumors with unknown EBV status. From 2005, more than 50% of tumors were tested for EBV.

**Table 1:** Characteristics of the study population subdivided by Epstein–Barr virus (EBV) status

	EBV status *N* (%)			
	Total population 1755 (100)	EBV positive 650 (37)	EBV negative 154 (9)	EBV unknown 951 (54)
Age	55 (9–97)	51 (9–97)	59 (9–84)	58 (10–88)
Sex				
Male	1256 (71)	483 (74)	99 (64)	674 (71)
Female	499 (28)	167 (26)	55 (36)	277 (29)
*T* stage				
*Tx*	88 (5)	20 (3)	4 (3)	64 (7)
*T*0	1 (< 1)	1 (< 1)	0 (0)	0 (0)
*T*1/*T*2	798 (45)	341 (52)	63 (41)	394 (41)
*T*3/*T*4	868 (50)	288 (44)	87 (56)	493 (52)
*N* stage				
*Nx*	71 (4)	12 (2)	5 (3)	54 (6)
*N*0	377 (21)	107 (17)	46 (30)	224 (24)
*N*1	396 (22)	168 (26)	44 (29)	184 (19)
*N*2	721 (41)	278 (43)	49 (32)	394 (41)
*N*3	190 (11)	75 (12)	10 (6)	95 (10)
*M* stage				
*M*0/*x*	1657 (94)	620 (95)	140 (91)	897 (94)
*M*1	98 (6)	30 (5)	14 (9)	54 (6)
Stage				
I	91 (5)	38 (6)	11 (7)	42 (5)
II	270 (15)	113 (18)	23 (15)	134 (14)
III	467 (27)	215 (33)	49 (32)	203 (21)
IV	894 (51)	275 (42)	68 (44)	551 (58)
Unknown	33 (2)	9 (1)	3 (2)	21 (2)
Pathological classification				
WHO type I	208 (12)	2 (< 1)	55 (36)	153 (16)
WHO type II	347 (20)	131 (20)	61 (41)	151 (16)
WHO type III	981 (56)	481 (74)	25 (16)	475 (50)
Basaloid	12 (< 1)	3 (< 1)	0 (0)	9 (1)
NOS	207 (12)	31 (5)	12 (7)	164 (17)

**Table 2:** Characteristics of the total study population subdivided by year of diagnosis

	Year of diagnosis *N* (%)						
	Total population 1755	1989–1993 283	1994–1998 240	1999–2003 273	2004–2008 296	2009–2013 319	2014–2018 343
Mean age (range)	55 (9–97)	55 (11–88)	56 (14–86)	54 (9–88)	55 (10–87)	54 (9–87)	56 (11–97)
Sex							
Male	1256 (71)	208 (73)	176 (73)	203 (74)	206 (70)	224 (70)	239 (70)
Female	499 (28)	75 (27)	64 (27)	70 (25)	90 (30)	96 (30)	104 (30)
*T* stage							
*Tx*	88 (5)	24 (9)	17 (7)	23 (9)	11 (4)	9 (3)	2 (1)
*T*0	1 (< 1)	0 (0)	0 (0)	0 (0)	0 (0)	0 (0)	1 (< 1)
*T*1/*T*2	798 (45)	114 (40)	81 (34)	135 (49)	151 (51)	155 (48)	162 (47)
*T*3/*T*4	868 (50)	145 (51)	142 (59)	115 (42)	134 (45)	156 (49)	176 (52)
*N* stage							
*Nx*	71 (4)	20 (8)	12 (6)	15 (5)	9 (3)	10 (3)	5 (1)
*N*0	377 (21)	71 (26)	57 (24)	57 (21)	61 (21)	62 (20)	69 (20)
*N*1	396 (22)	35 (13)	29 (13)	64 (23)	78 (26)	87 (27)	103 (30)
*N*2	721 (41)	123 (44)	107 (45)	105 (38)	119 (40)	127 (40)	122 (36)
*N*3	190 (11)	24 (9)	27 (12)	32 (12)	29 (10)	34 (10)	44 (13)
*M* stage							
*M*0/*x*	1657 (94)	273 (96)	231 (96)	255 (94)	280 (95)	298 (93)	312 (92)
*M*1	98 (6)	10 (4)	9 (4)	15 (6)	15 (5)	21 (7)	28 (8)
Tumor stage							
I	91 (5)	12 (4)	10 (4)	11 (4)	13 (4)	16 (5)	19 (8)
II	270 (15)	16 (6)	11 (5)	63 (23)	66 (22)	57 (18)	57 (17)
III	467 (27)	43 (15)	33 (14)	86 (31)	90 (31)	113 (35)	102 (30)
IV	894 (51)	202 (71)	183 (76)	103 (38)	114 (42)	130 (41)	152 (44)
Unknown	33 (2)	10 (4)	3 (1)	10 (4)	3 (1)	4 (1)	3 (1)
Pathological classification							
WHO type I	208 (12)	41 (14)	32 (13)	24 (10)	27 (9)	46 (15)	38 (11)
WHO type II	347 (20)	33 (11)	38 (16)	41 (14)	62 (20)	70 (22)	103 (30)
WHO type III	981 (56)	175 (62)	128 (53)	166 (61)	179 (61)	174 (54)	159 (46)
Basaloid	12 (< 1)	0 (0)	2 (1)	4 (1)	2 (1)	2 (1)	2 (1)
NOS	207 (12)	34 (13)	40 (17)	39 (14)	26 (9)	27 (8)	41 (12)
EBV status							
Positive	650 (37)	2 (1)	21 (9)	82 (30)	149 (50)	184 (57)	212 (62)
Negative	154 (9)	2 (1)	6 (3)	11 (4)	23 (8)	44 (15)	68 (20)
Unknown	952 (54)	279 (98)	213 (89)	180 (66)	124 (41)	92 (28)	63 (18)

*EBV* Epstein–Barr virus, *NOS* not otherwise specified, *WHO* World Health Organization

### Incidence

**Fig. 2:**
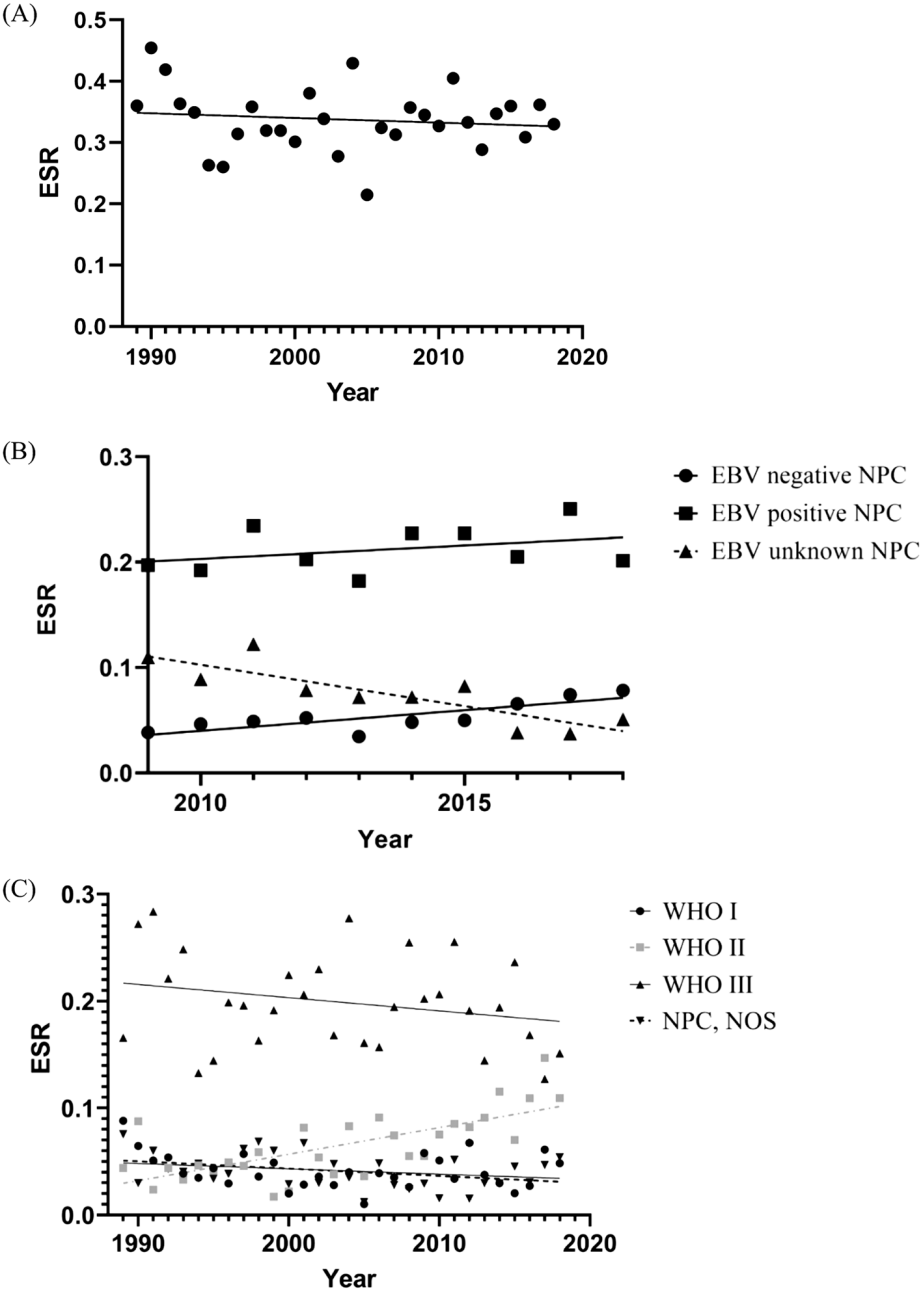
Incidence of nasopharyngeal cancer(NPC) in the Netherlands over time. **A** Incidence of the total study population. The European standardized rate (ESR) for each year is represented by a dot. A linear regression model was fitted to estimate annual percentage change (APC). **B** Incidence of NPC according to Epstein–Barr virus (EBV) status. The ESR value of each subgroup for each year is represented by a dot, triangle or square. A linear regression model was fitted to estimate APC. **C** Incidence of NPC according to pathological classification. The ESR value for each year is represented by a dot, triangle or square. A linear regression model was fitted to estimate APC. Basaloid tumors were excluded due to the limited number of cases. *WHO* World Health Organization, *NOS* not otherwise specified

The absolute number of NPC diagnoses increased from 283 in 1989–1993 to 343 in 2014–2018. However, the ESR varied between 0.22 and 0.46 per 100,000 (Fig. 2a and Supplementary Table S2, Appendix A) and the APC equaled − 0.2%, (95% CI − 0.9; 0.5) over the total time period, indicating that there is no statistically significant change in ESR over time.

Since EBV status in early years was often unknown, we restricted analyses for the incidence by EBV status to the last 10 years. The incidence of EBV-positive NPC did not change over the last decade (APC 1.2 (95% CI − 1.3; 3.8; *p* 0.31). We found a significant increase in EBV-negative tumors with 7.1% (95% CI 2.5; 11.9; *p* < 0.01) per year between 2009 and 2018 and a decrease in tumors with an unknown EBV status, with an APC of − 10.7 (95% CI − 15.7; − 5.7; *p* < 0.01, Fig. 2b and Supplementary Table S2, Appendix A).

Over the entire time period, no significant change in incidence rates of WHO type I NPC (APC: − 1.1; 95 CI − 2.9; 0.7; *p* = 0.26) or WHO type III NPC (APC: − 0.6; 95% CI − 1.5; 0.3; *p* 0.21) was observed. However, an increase in WHO type II NPC with an APC of 3.8 (95% CI 2.2; 5.5; *p* < 0.001) was found. The incidence of the basaloid subtype was too low to allow for trend analysis of incidence over time. There was no significant change in incidence of NPC NOS, with an APC of − 1.6 (95% CI − 3.5; 0.2; *p* 0.09, Fig. 2c and Supplementary Table S2, Appendix A).

### Survival

**Fig. 3:**
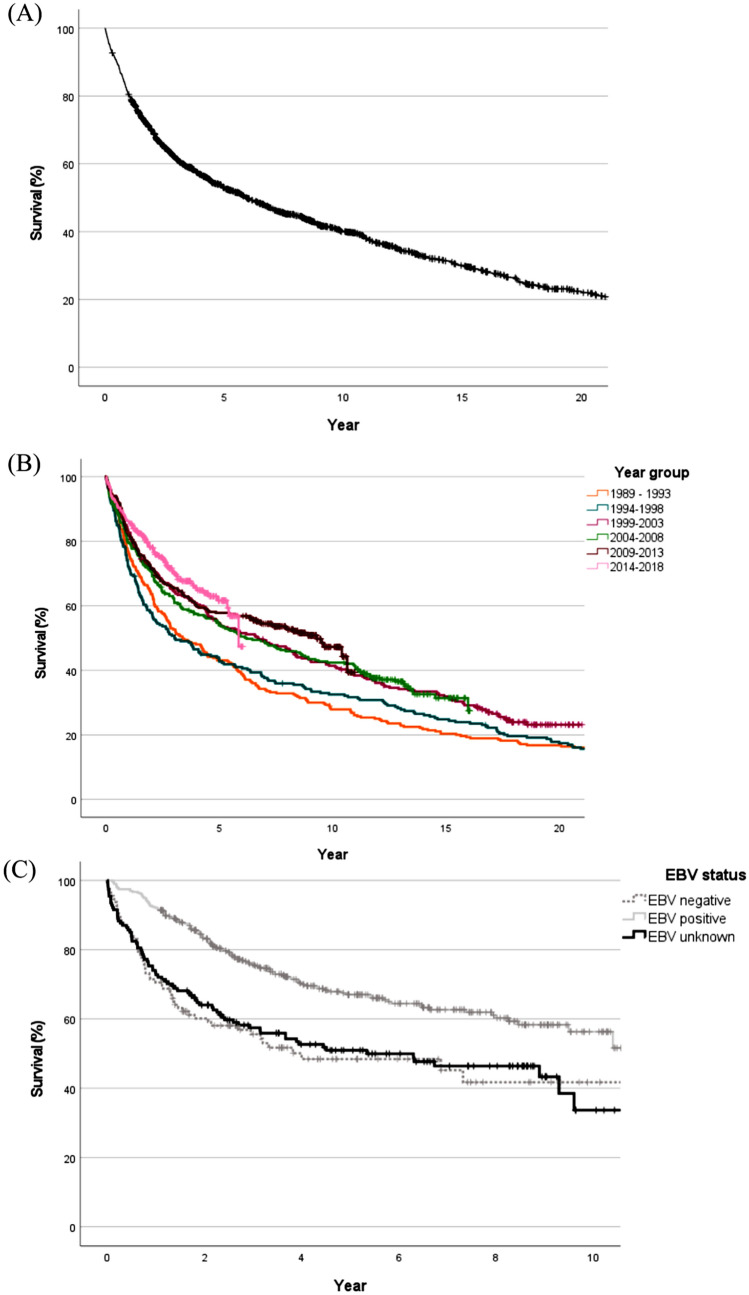
Survival curves. **A** Kaplan–Meier survival curve for overall survival of all patients diagnosed with nasopharyngeal carcinoma (NPC) in the Netherlands between 1989 and 2018. **B** Kaplan–Meier survival curve of all patients, according to year of diagnosis. **C** Kaplan–Meier survival curves of patients diagnosed between 2009 and 2018 according to Epstein–Barr virus (EBV) status

Median survival for the total population was 5.9 years (95% CI 5.1–6.8, Fig. 3a and Table S3, Appendix A). Five-year relative survival was 56% (95% CI 5.1–6.8) for the total cohort. (Table S4, Appendix A). We found a better survival in patients diagnosed in 1999 or thereafter, compared to patients diagnosed earlier; the hazard ratio (HR) of dying for year groups after 1998 was 0.8 (95% CI 0.6–0.9) for 1999–2003, 0.8 (95% CI 0.6–0.9) for 2004–2008, 0.6 (95% CI 0.5–0.8) for 2009–2013, and 0.6 (95% CI 0.5–0.7) for 2014–2018 (Fig. 3b and Table S5, Appendix A). However, period of diagnosis was not an independent risk factor for relative excess risk of dying. Negative EBV status, WHO type I histology, higher stage, and higher age at diagnosis were independently associated with a higher relative excess risk of dying. (Table S6, Appendix A).

## Discussion

The aim of this study was to analyze trends in incidence and survival of NPC over time in the Netherlands which is a non-endemic region. We found no significant change in the overall incidence of NPC over 30 years, nor in EBV-positive NPC over the last decade. An increase of EBV-negative tumors over time was paralleled by a decrease of NPC diagnoses with an unknown EBV status. The incidence of WHO type III and WHO type I NPC remained stable but the incidence WHO type II tumors has increased over time.

The stable ESR of NPC deviated from some other studies in non-endemic regions where a decrease in incidence of NPC was demonstrated (Tang et al. 2016; Argirion et al. 2019). However, in line with our results, data from Scotland showed a stable ESR over the period 1975–2001 (Anandan et al. 2008). In endemic regions, mostly a decrease in incidence was shown (Tang et al. 2016; Lee et al. 2003; Hsu et al. 2006), although data from southeast China suggest a stable incidence between 1987 and 2011 (Xie et al. 2015).

We found that the ESR of EBV-positive NPC cases remained stable over the last 10 years. The increase in EBV-negative tumors that we found reflects a rise in EBV testing over time as shown by a parallel decrease in cases with an EBV-unknown status. Because the EBV status was not frequently reported in early years, we cannot draw conclusions about the true incidence of EBV-negative tumors. Although evaluation of all pathology reports improved the reliability of the diagnosis and of histological classification, a further improvement would be to perform EBV testing retrospectively on archival tumor tissue.

An increase in incidence of WHO type II NPC was also found in the US (Argirion et al. 2019). Compared to an earlier nationwide study from the Netherlands, we found a lower incidence of WHO type I NPC and a higher incidence of NPC NOS cases (Arnold et al. 2013). This may be due to the fact that we had access to the pathology reports and used this for pathological classification. If keratinization was not mentioned in the pathology description of the tumor and therefore no certain distinction between WHO type I and II could be made, cases were classified as NOS. If these cases would have been classified as WHO type I NPC, our results would be more in line with other reports (Arnold et al. 2013; Argirion et al. 2019).

Survival was better for patients diagnosed after 1998, but the period of diagnosis was not an independent risk factor. This suggests that the improved survival after 1998 was associated with changes in the characteristics of the patient population. We found indeed a decrease in stage IV tumors after 1998; however, this could be related to the introduction of the TNM5. Invasion of the bone of the skull was classified as T4 in the TNM4 and as T3 thereafter. Similarly single or multiple ipsilateral lymph nodes < 6 cm were classified as N2a-b in the TNM4 and as N1 thereafter. There was no decrease in the proportion of patients with distant metastasis. Another explanation could be a shift toward histological subtypes with a more favorable prognosis. We indeed found an increase of WHO type II NPC, which is described in the literature and shown in our study to have a more favorable prognosis than WHO type I and NPC NOS (Arnold et al. 2013; Argirion et al. 2019). As in previous reports, we found that patients with EBV-positive and WHO type III tumors have the highest overall survival (Tang et al. 2016; Argirion et al. 2019).

Treatment regimens in non-endemic regions are mostly based on studies from endemic regions with non-keratinizing NPC (Bossi et al. 2021). Because there is a difference in incidence of subtypes between endemic and non-endemic regions, clinical trials in non-endemic regions, especially for non-WHO type III disease, are needed. Furthermore, evidence is emerging that HPV plays a role in NPC, and data on outcome are scarce (Robinson et al. 2013; Simon et al. 2020; Huang et al. 2022). In conclusion, our study shows a stable incidence of NPC in the Netherlands over the last 30 years, with a significant increase in WHO type II NPC, a stable incidence in EBV-positive tumors over the last 10 years. Survival was better after 1998, presumably as a consequence of a shift in tumor characteristics.

### Supplementary Information

Below is the link to the electronic supplementary material.

Supplementary file1 (DOCX 56 KB)

## Data Availability

Upon reasonable request to the corresponding author, (1) the data dictionary, (2) syntaxes, and (3) deidentified participant data supporting the findings in this study (based on a signed data access agreement), can be made available.

## Abbreviations

APC: Annual percentage change
ASR: Age standardized incidence rate
EBV: Epstein–Barr virus
ESR: European Standardized incidence rate
NOS: Not otherwise specified
NPC: Nasopharyngeal carcinoma
PALGA: The nationwide network and registry of histopathology and cytopathology in the Netherlands
WHO: World Health Organization
