# Supplementary material for: Nasopharyngeal carcinoma: nationwide trends in subtype-specific incidence and survival over 3 decades in a non-endemic area
Source: J Cancer Res Clin Oncol. 2024 Jan 29;150(2):49. doi: 10.1007/s00432-023-05547-8 (PMC10824861; doi:10.1007/s00432-023-05547-8)
Supplement: Supplementary file 1 — Supplementary file1 (DOCX 56 KB) [file 432_2023_5547_MOESM1_ESM.docx]

**Supplementary data**

Table S1A Differences in T, N and M classifications between editions of the TNM classification of malignant tumours for nasopharyngeal carcinoma according to the Union for International Cancer Control

|  | TNM 4 + TNM 4 2^nd^ revision. | TNM 5 | TNM 6 | TNM 7 | TNM 8 |
| --- | --- | --- | --- | --- | --- |
|  |  |  |  |  |  |
|  |  |  |  |  |  |
| T1 | Limited to 1 subsite | Limited to nasopharynx | Limited to Nasopharynx | Limited to nasopharynx, oropharynx or nasal cavity | Limited to Nasopharynx, oropharynx or nasal cavity without parapharyngeal extension |
| T2 | Invading 1 subsite | Invading the oropharynx and/ or nasal fossa |  | Parapharyngeal extension | Parapharyngeal extension, adjacent soft tissue involvement (medial pterygoid, lateral pterygoid, prevertebral muscles) |
| T2a |  | Without parapharyngeal extension | Invading the Oropharynx and/or nasal cavity |  |  |
| T2b |  | With parapharyngeal extension | With parapharyngeal extension |  |  |
| T3 | Invading oropharynx and or nasal cavity | Invading bony structures and/or paranasal sinuses | Invading bony structures and/or paranasal sinuses | Invading bony structures and/or paranasal sinuses | Invading bony structures (skull base, cervical vertebra) and/or paranasal sinuses |
| T4 | Invading skull and/or cranial nerves | Intracranial extension and/or involvement of cranial nerves, infratemporal fossa. Hypopharynx, or orbit | Intracranial extension and/or cranial nerves, infratemporal fossa hypopharynx, orbit or masticatory space | Intracranial extension and/or cranial nerves, hypopharynx, orbit or infratemporal fossa/ masticatory space | Intracranial extension, cranial nerve, hypopharynx, orbit, extensive soft tissue involvement (beyond the lateral surface of the lateral pterygoid muscle), parotid gland |
| N0 | No lymph node metastasis | No lymph node metastasis | No regional lymph node metastasis | No regional lymph node metastasis | No regional lymph node metastasis |
| N1 | Single ipsilateral lymph node (<3 cm) | Ipsilateral lymph node metastasis (<6 cm) | Unilateral node(s), ≤6cm in greatest dimension, above the supraclavicular fossa | Unilateral cervical and/or unilateral or bilateral retropharyngeal node(s), ≤6cm in greatest dimension, above the supraclavicular fossa | Unilateral cervical, unilateral or bilateral retropharyngeal lymph nodes, above the caudal border of cricoid cartilage; ≤6 cm |
| N2 |  | Bilateral lymph node metastasis (<6 cm) | Bilateral node(s), ≤6cm in greatest dimension, above the supraclavicular fossa | Bilateral cervical node(s),≤6cm in greatest dimension, above the supraclavicular fossa | Bilateral metastasis in lymph node(s), 6 cm or less in greatest dimension, above the caudal border of cricoid cartilage |
| N2a | Single ipsilateral lymph node (3-6 cm) |  |  |  |  |
| N2b | Multiple ipsilateral lymph nodes (<6 cm) |  |  |  |  |
| N2c | Bilateral or contralateral lymph nodes (<6 cm) |  |  |  |  |
| N3 | Lymph node metastasis (>6 cm) | Lymph node metastasis (>6 cm) or extension to the supraclavicular fossa | >6cm, or in supraclavicular fossa | >6cm, or in supraclavicular fossa | >6 cm and/or below caudal border of cricoid cartilage (regardless of laterality) |
| N3a |  |  | > 6 cm | > 6 cm |  |
| N3b |  |  | Extension to supraclavicular fossa | Extension to supraclavicular fossa |  |
| M0 | No distant metastasis | No distant metastasis | No distant metastasis | No distant metastasis | No distant metastasis |
| M1 | Distant metastasis | Distant metastasis | Distant metastasis | Distant metastasis | Distant metastasis |

EBV: Epstein-Barr virus.

Table S1B Differences in the classification of stage between editions of the TNM classification of malignant tumours for nasopharyngeal carcinoma according to the Union for International Cancer Control

|  | TNM 4 + TNM 4 2^nd^ revision. | TNM 5 | TNM 6 | TNM 7 | TNM 8 |
| --- | --- | --- | --- | --- | --- |
| Stage I | T1N0M0 | T1N0M0 | T1N0M0 | T1N0M0 | T1 N0 M0 |
| Stage II | T2N0M0 |  |  | T1N1M0, T2N0-1M0 | T0-1 N1 M0,  T2 N0-1 M0 |
| Stage IIa |  | T2aN0M0 | T2aN0M0 |  |  |
| Stage IIb |  | T1-2aN1M0, T2bN0-1M0 | T1-2aN1M0, T2bN0-1M0 |  |  |
| Stage III | T3N0M0 or T1-3N1M0 | T1-2N2M0 or T3N0-2M0 | T1-2bN2M0, T3N0-2M0 | T1-2N2M0, T3N0-2M0 | T3N0-2 M0, T1-2 N2 M0 |
| Stage IV | T4 (any N) or N2-N3 (any T) or M1 (any T any N) |  |  |  |  |
| Stage IVA |  | T4N0-2M0 | T4N0-2M0 | T4N0-2M0 | T4 or N3 M0 |
| Stage IVB |  | Any T, N3M0 | Any T, N3M0 | Any T, N3M0 | Any T, any N M1 |
| Stage IVC |  | Any T, Any M, M1 | Any T, any N, M1 | Any T, any N, M1 |  |

EBV: Epstein-Barr virus.

Supplementary Table S2: Annual percentage change in incidence

|  | APC | 95 CI low | 95% CI high | P value |
| --- | --- | --- | --- | --- |
| Total | -0.2 | -0.9 | 0.5 | 0.596 |
| EBV negative | 7.1 | 2.5 | 11.9 | 0.007 |
| EBV positive | 1.2 | -1.3 | 3.8 | 0.314 |
| EBV unknown | -10.7 | -15.7 | -5.3 | 0.002 |
| WHO type I | -1.1 | -2.9 | 0.8 | 0.256 |
| WHO type II | 3.8 | 2.2 | 5.5 | < 0.001 |
| WHO type III | -0.6 | -1.5 | 0.3 | 0.203 |
| NOS | -1.6 | -3.5 | 0.2 | 0.085 |

Annual percentage change (APC) in incidence of nasopharyngeal cancer by Epstein-Barr virus (EBV) status was calculated over the period 2009-2018. The other cohorts were analyzed over the full period, 1989-2018. EBV: Epstein-Barr virus, WHO: world health organization, NOS: not otherwise specified.

Supplementary Table S3. Survival analysis for subgroups

| Group | Median survival  (years) | 95% CI low | 95% CI high |
| --- | --- | --- | --- |
| Total | 5.9 | 5.1 | 6.8 |
| 1989-1993 | 3.4 | 2.4 | 4.3 |
| 1994-1998 | 3.0 | 1.8 | 4.2 |
| 1999-2003 | 6.7 | 4.7 | 8.7 |
| 2004-2008 | 6.2 | 4.1 | 8.3 |
| 2009-2013 | 9.3 | 7.2 | 11.4 |
| 2014-2018 | Not reached |  |  |
| EBV positive | 10.6 | 9.1 | 12.1 |
| EBV negative | 4.0 | 0.8 | 7.2 |
| EBV unknown | 5.3 | 1.6 | 9.1 |
| WHO type I | 5.0 | 4.0 | 6.1 |
| WHO type II | 9.8 | 8.6 | 11.0 |
| WHO type III | 12.2 | 11.4 | 13.1 |
| NOS | 7.8 | 6.3 | 9.3 |
| Stage I | 13.0 | 11.2 | 14.7 |
| Stage II | 10.9 | 9.9 | 12.0 |
| Stage III | 9.8 | 7.5 | 12.0 |
| Stage IV | 3.0 | 2.5 | 3.6 |

Median overall survival was calculated with the Kaplan-Meier analysis for patients diagnosed with nasopharyngeal cancer between 1989-2018 subdivided in 5 equal year groups, by EBV status between 2009-2018, and by histopathological classification for the entire period. Basaloid tumors were excluded due to the limited number of cases. EBV: Epstein-Barr virus NOS: not otherwise specified, WHO: world health organization.

Supplementary Table S4: 5-year relative survival

|  | 5-year relative survival | 95% CI low | 95% CI high |
| --- | --- | --- | --- |
| Total | 56% | 54% | 59% |
| Year of diagnosis |  |  |  |
| 1989-1993 | 48% | 41% | 54% |
| 1994-1998 | 46% | 39% | 52% |
| 1999-2003 | 58% | 52% | 64% |
| 2004-2008 | 57% | 51% | 63% |
| 2009-2013 | 61% | 55% | 66% |
| 2014-2018 | 65% | 58% | 72% |
| EBV status 2009-2018 |  |  |  |
| EBV Positive | 70% | 64% | 75% |
| EBV Negative | 51% | 40% | 61% |
| EBV Unknown | 55% | 45% | 63% |
| Pathological classification |  |  |  |
| WHO type I | 33% | 26% | 40% |
| WHO type II | 62% | 56% | 67% |
| WHO type III | 62% | 58% | 65% |
| NOS | 46% | 38% | 53% |
| Stage |  |  |  |
| Stage I | 84% | 72% | 92% |
| Stage II | 76% | 69% | 81% |
| Stage III | 65% | 60% | 70% |
| Stage IVM0 | 48% | 44% | 52% |
| M1 | 10% | 5% | 17% |
| Unknown | 36% | 19% | 54% |
| Sex |  |  |  |
| Male | 55% | 52% | 58% |
| Female | 58% | 53% | 63% |
| Age at diagnosis |  |  |  |
| 0-17 | 78% | 62% | 88% |
| 18-44 | 73% | 68% | 78% |
| 45-54 | 61% | 55% | 66% |
| 55-64 | 57% | 52% | 62% |
| 65-74 | 44% | 38% | 50% |
| 75+ | 23% | 15% | 32% |

Relative 5-year survival was calculated using the Ederer II method for relative survival for patients diagnosed with nasopharyngeal cancer between 1989-2018 subdivided in 5 equal year groups, by EBV status between 2009-2018, and by histopathological classification for the entire period. Basaloid tumors were excluded due to the limited number of cases. EBV: Epstein-Barr virus, NOS: not otherwise specified, WHO: world health organization.

Supplementary Table S5: Univariable analysis of hazard ratios for dying

| Year of diagnosis | HR | 95% CI low | 95% CI high |
| --- | --- | --- | --- |
| 1989-1993 | 1 |  |  |
| 1994-1998 | 1.0 | 0.8 | 1.2 |
| 1999-2003 | 0.8 | 0.6 | 0.9 |
| 2004-2008 | 0.8 | 0.6 | 0.9 |
| 2009-2013 | 0.6 | 0.5 | 0.8 |
| 2014-2018 | 0.6 | 0.5 | 0.7 |
| EBV status 2009-2018 |  |  |  |
| EBV Positive | 1 |  |  |
| EBV Negative | 2.1 | 1.5 | 2.8 |
| EBV unknown | 1.8 | 1.4 | 2.4 |
| Pathological classification |  |  |  |
| WHO type I | 2.4 | 2.0 | 2.9 |
| WHO type II | 1.2 | 1.0 | 1.4 |
| WHO type III | 1 |  |  |
| NOS | 1.7 | 1.4 | 2.0 |

Univariable analysis of hazard ratios calculated with the cox regression analysis for patients diagnosed with nasopharyngeal cancer between 1989-2018 subdivided in 5 equal year groups, or subdivided by EBV status for 1998-2018, or histopathological classification for the entire period. Basaloid tumors were excluded due to the limited number of cases. EBV: Epstein-Barr virus, HR: hazard ratio, WHO: world health organization, NOS: not otherwise specified.

Supplementary Table S6A: Univariable and multivariable analysis of relative excess risk of dying

|  | Univariable analysis | | | Multivariable analysis | | |
| --- | --- | --- | --- | --- | --- | --- |
|  | RER | 95% CI low | 95% CI high | RER | 95% CI low | 95% CI high |
| Year of diagnosis |  |  |  |  |  |  |
| 1989-1993 | 1.0 |  |  | 1.0 |  |  |
| 1994-1998 | 1.0 | 0.8 | 1.2 | 1.2 | 0.9 | 1.5 |
| 1999-2003 | 0.7 | 0.5 | 0.8 | 0.9 | 0.7 | 1.2 |
| 2004-2008 | 0.7 | 0.5 | 0.9 | 1.0 | 0.8 | 1.2 |
| 2009-2013 | 0.6 | 0.5 | 0.8 | 0.8 | 0.6 | 1.0* |
| 2014-2018 | 0.8 | 0.6 | 1.0 | 0.8 | 0.7 | 1.1 |
| Pathological classification |  |  |  |  |  |  |
| WHO type I | 2.8 | 2.3 | 3.4 | 2.0 | 1.6 | 2.4 |
| WHO type II | 1.1 | 0.9 | 1.4 | 1.0 | 0.9 | 1.3 |
| WHO type III | 1.0 |  |  | 1.0 |  |  |
| NOS | 1.9 | 1.6 | 2.4 | 1.7 | 1.4 | 2.1 |
| Stage |  |  |  |  |  |  |
| Stage I | 1.0 |  |  | 1.0 |  |  |
| Stage II | 1.7 | 0.9 | 3.3 | 2.2 | 1.1 | 4.1 |
| Stage III | 2.4 | 1.3 | 4.5 | 3.6 | 1.9 | 6.7 |
| Stage IVM0 | 4.5 | 2.5 | 8.2 | 6.1 | 3.3 | 11.2 |
| M1 | 17.9 | 9.5 | 33.7 | 26.2 | 13.9 | 49.6 |
| Unknown | 6.1 | 2.9 | 12.8 | 7.0 | 3.4 | 14.8 |
| Sex |  |  |  |  |  |  |
| Male | 1.0 |  |  | 1.0 |  |  |
| Female | 0.9 | 0.7 | 1.0 | 0.9 | 0.8 | 1.1 |
| Age at diagnosis |  |  |  |  |  |  |
| 0-17 | 0.6 | 0.3 | 1.2 | 0.4 | 0.2 | 0.8 |
| 18-44 | 1.0 |  |  | 1.0 |  |  |
| 45-54 | 1.7 | 1.3 | 2.1 | 1.6 | 1.2 | 2.0 |
| 55-64 | 2.1 | 1.7 | 2.6 | 1.9 | 1.5 | 2.4 |
| 65-74 | 3.2 | 2.5 | 3.4 | 2.7 | 4.3 | 3.5 |
| 75+ | 7.2 | 5.5 | 9.3 | 6.8 | 5.2 | 9.0 |

Univariable analysis and multivariable analysis of relative excess risk calculated with Poisson regression modeling for patients diagnosed with nasopharyngeal cancer between 1989-2018 subdivided in 5 equal year groups, or histopathological classification, or stage, or sex, age group for the entire period. Basaloid tumors were excluded due to the limited number of cases. RER: relative excess risk, WHO: world health organization, NOS: not otherwise specified. *Not statistically significant

Supplementary Table S6B: Univariable and multivariable analysis of relative excess risk of dying for time period 2009-2018

|  | Univariable analysis  2009-2018 | | | Multivariable analysis 2009-2018 | | |
| --- | --- | --- | --- | --- | --- | --- |
|  | RER | 95% CI low | 95% CI high | RER | 95% CI low | 95% CI high |
| EBV status 2009-2018 |  |  |  |  |  |  |
| EBV Positive | 1.0 |  |  | 1.0 |  |  |
| EBV Negative | 2.4 | 1.7 | 3.4 | 2.0 | 1.4 | 2.8 |
| EBV Unknown | 1.9 | 1.4 | 2.5 | 1.4 | 1.0 | 1.9 |
| Stage |  |  |  |  |  |  |
| Stage I & II | 1.0 |  |  | 1.0 |  |  |
| Stage III | 2.2 | 1.3 | 3.7 | 2.7 | 1.6 | 4.5 |
| Stage IV | 3.8 | 2.4 | 6.2 | 4.5 | 2.8 | 7.2 |
| Sex |  |  |  |  |  |  |
| Male | 1.0 |  |  | 1.0 |  |  |
| Female | 0.9 | 0.7 | 1.2 | 0.9 | 0.7 | 1.2 |
| Age at diagnosis |  |  |  |  |  |  |
| 0-44 | 1.0 |  |  | 1.0 |  |  |
| 45-64 | 2.0 | 1.3 | 3.0 | 1.7 | 1.1 | 2.6 |
| 65+ | 4.1 | 2.7 | 6.2 | 3.9 | 2.5 | 5.9 |

Univariable analysis and multivariable analysis of relative excess risk calculated with Poisson regression modeling for patients diagnosed with nasopharyngeal cancer between 2009-2018 subdivided by EBV status, stage, or sex or age at diagnosis. Basaloid tumors were excluded due to the limited number of cases. Stage unknown cases were excluded due to limited number of cases. EBV: Epstein-Barr virus, RER: relative excess risk.
